# Bearing vibration data collected under time-varying rotational speed conditions

**DOI:** 10.1016/j.dib.2018.11.019

**Published:** 2018-11-09

**Authors:** Huan Huang, Natalie Baddour

**Affiliations:** Department of Mechanical Engineering, University of Ottawa, Ottawa, Ontario, Canada

## Abstract

Vibration signal analysis is an important means for bearing fault detection/diagnosis and bearings often operate under time-varying rotational speed conditions. This data article contains vibration datasets collected from bearings with different health conditions under different time-varying speed conditions. The health conditions of the bearing include healthy, faulty with an inner race defect, and faulty with an outer race defect. The operating rotational speed conditions for the dataset include increasing speed, decreasing speed, increasing then decreasing speed, and decreasing then increasing speed. Mendeley Data, http://dx.doi.org/10.17632/v43hmbwxpm.1.

**Specifications table**

**Table:** 

Subject area	*Mechanical engineering*
More specific subject area	*Vibration, machine condition monitoring*
Type of data	*.mat file*
How data were acquired	*An accelerometer was used to collect the vibration data and an incremental encoder was used to collect the rotational speed data*
Data format	*Raw*
Experimental factors	*Two experimental settings: bearing health condition and varying speed condition*
Experimental features	*Both bearing health condition and varying speed condition will change the frequency characteristics of the bearing vibration data*
Data source location	*Ottawa, Canada*
Data accessibility	*Mendeley Data.*http://dx.doi.org/10.17632/v43hmbwxpm.1

**Value of the data**

• The data are collected from bearings operating under time-varying rotational speed conditions. This dataset differs from existing datasets in the literature that have been collected under constant speed condition.
• The collected data can be used to analyze the frequency characteristics of bearings of different health conditions under time-varying speed conditions.
• The data can also be applied to assess the effectiveness of any newly developed method for bearing fault diagnosis or condition monitoring under time-varying speed conditions.

## 1 Data

The data contain vibration signals collected from bearings under time-varying rotational speed conditions. The data can be employed to evaluate the effectiveness of methods developed for bearing fault diagnosis under time-varying speed conditions, such as the methods proposed in [1], [2], [3], [4].

## 2 Experimental design, materials and methods

### 2.1 Experimental set-up

**Fig. 1:**
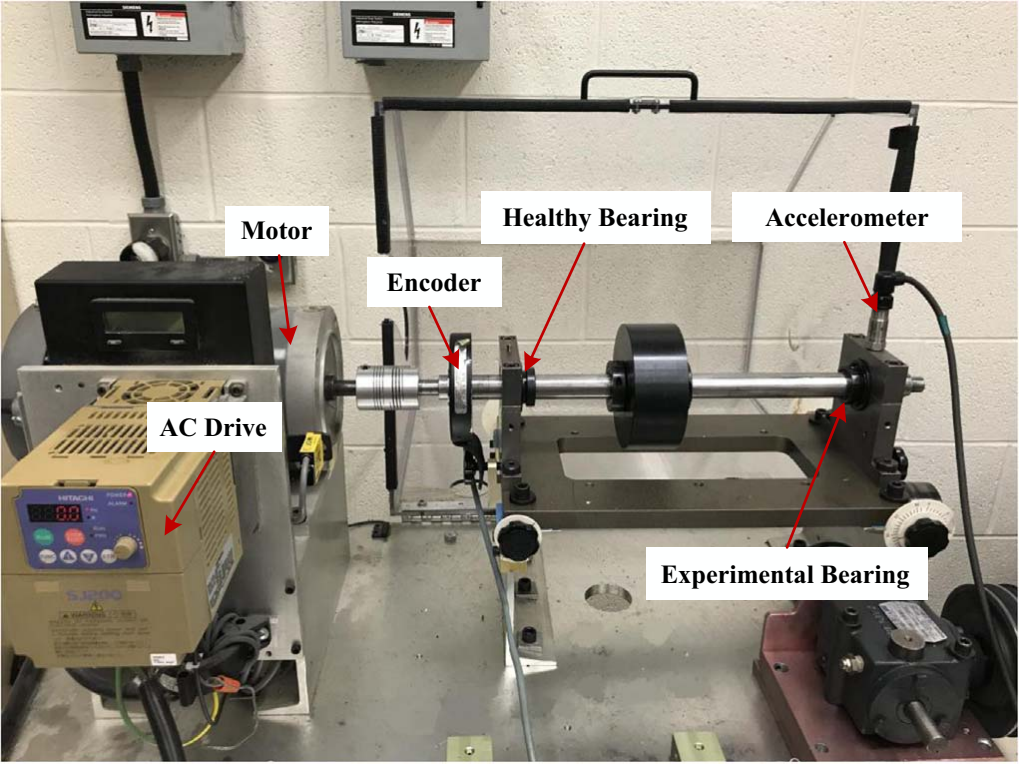
Experimental set-up.

Experiments are performed on a SpectraQuest machinery fault simulator (MFS-PK5M). The experimental set-up is shown in Fig. 1. The shaft is driven by a motor and the rotational speed is controlled by an AC drive. Two ER16K ball bearings are installed to support the shaft, the left one is a healthy bearing and the right one is the experimental bearing, which is replaced by bearings of different health conditions. An accelerometer (ICP accelerometer, Model 623C01) is placed on the housing of the experimental bearing to collect the vibration data. In addition, an incremental encoder (EPC model 775) is installed to measure the shaft rotational speed.

Bearing faults can be detected and diagnosed by observing the Fault Characteristic Frequency (FCF) in the frequency domain [5]. For each type of fault, it has a specific FCF, which is proportional to the operating rotational frequency, and the coefficient is determined by the bearing structural parameters. The structural parameters of the bearings used in the experiments are given in Table 1. According to the parameters of the bearings, the FCF coefficient of the bearing inner race is 5.43 [5], i.e. Ball-Pass Frequency of Inner-race (BPFI) is equal to the product of the FCF coefficient (5.43) and the shaft rotational frequency *f*_r_, i.e. BPFI = 5.43 *f*_r_. Similarly, the FCF of the bearing outer race, i.e. Ball-Pass Frequency of Outer-race (BPFO) = 3.57 *f*_r_.

**Table 1:** Parameters of bearings.

Bearing type	Pitch diameter	Ball diameter	Number of balls	BPFI	BPFO
ER16K	38.52 mm	7.94 mm	9	5.43*f*_r_	3.57*f*_r_

### 2.2 Data acquisition settings

The data are acquired by the NI data acquisition boards (NI USB-6212 BNC). The accelerometer measures vibration data and the encoder measures rotational speed data. Each sampled dataset contains two channels, and both are saved in one.mat file. ‘Channel_1’ is the vibration data measured by the accelerometer and ‘Channel_2’ is the rotational speed data measured by the encoder.

#### 2.2.1 Sampling frequency and length

In all the experiments, both ‘Channe_1’ and ‘Channel_2’, signals are sampled at 200,000 Hz and the sampling duration is 10 s.

#### 2.2.2 Datasets design

There are 36 datasets in total available at http://dx.doi.org/10.17632/v43hmbwxpm.1. The numbering of the dataset is given in Table 2. For each dataset, there are two experimental settings: bearing health condition and varying speed condition. The health conditions of the bearing include (i) healthy, (ii) faulty with an inner race defect, and (iii) faulty with an outer race defect. The operating rotational speed conditions are (i) increasing speed, (ii) decreasing speed, (iii) increasing then decreasing speed, and (iv) decreasing then increasing speed. Therefore, there are 12 different cases for the setting. To ensure the authenticity of the data, 3 trials are collected for each experimental setting which results in 36 datasets in total.

**Table 2:** Dataset numbering.

Bearing Health conditions	Speed varying conditions			
	Increasing speed	Decreasing speed	Increasing then decreasing speed	Decreasing then increasing speed
Healthy	H-A-1	H-B-1	H-C-1	H-D-1
	H-A-2	H-B-2	H-C-2	H-D-2
	H-A-3	H-B-3	H-C-3	H-D-3
Faulty (inner race fault)	I-A-1	I-B-1	I-C-1	I-D-1
	I-A-2	I-B-2	I-C-2	I-D-2
	I-A-3	I-B-3	I-C-3	I-D-3
Faulty (outer race fault)	O-A-1	O-B-1	O-C-1	O-D-1
	O-A-2	O-B-2	O-C-2	O-D-2
	O-A-3	O-B-3	O-C-3	O-D-3

To give a better idea of the collected datasets, the detailed operating conditions of the datasets numbered in Table 2 are given as follows (the data are all sampled at 200,000 Hz and the sampling duration is 10 s).

• Dataset H-A-1: the vibration data are collected from a healthy bearing and the operating rotational speed is increasing from 14.1 Hz to 23.8 Hz.
• Dataset H-A-2: the vibration data are collected from a healthy bearing and the operating rotational speed is increasing from 14.1 Hz to 29.0 Hz.
• Dataset H-A-3: the vibration data are collected from a healthy bearing and the operating rotational speed is increasing from 15.2 Hz to 26.7 Hz.
• Dataset H-B-1: the vibration data are collected from a healthy bearing and the operating rotational speed is decreasing from 28.9 Hz to 13.7 Hz.
• Dataset H-B-2: the vibration data are collected from a healthy bearing and the operating rotational speed is decreasing from 25.7 Hz to 11.6 Hz.
• Dataset H-B-3: the vibration data are collected from a healthy bearing and the operating rotational speed is decreasing from 28.6 Hz to 13.9 Hz.
• Dataset H-C-1: the vibration data are collected from a healthy bearing and the operating rotational speed is increasing from 14.7 Hz to 25.3 Hz, then decreasing to 21.0 Hz.
• Dataset H-C-2: the vibration data are collected from a healthy bearing and the operating rotational speed is increasing from 14.4 Hz to 24.0 Hz, then decreasing to 18.7 Hz.
• Dataset H-C-3: the vibration data are collected from a healthy bearing and the operating rotational speed is increasing from 15.4 Hz to 24.8 Hz, then decreasing to 19.1 Hz.
• Dataset H-D-1: the vibration data are collected from a healthy bearing and the operating rotational speed is decreasing from 24.2 Hz to 14.8 Hz, then increasing to 20.6 Hz.
• Dataset H-D-2: the vibration data are collected from a healthy bearing and the operating rotational speed is decreasing from 24.6 Hz to 14.0 Hz, then increasing to 18.6 Hz.
• Dataset H-D-3: the vibration data are collected from a healthy bearing and the operating rotational speed is decreasing from 26.0 Hz to 16.9 Hz, then increasing to 23.2 Hz.
• Dataset I-A-1: the vibration data are collected from a faulty bearing with an inner race defect and the operating rotational speed is increasing from 12.5 Hz to 27.8 Hz.
• Dataset I-A-2: the vibration data are collected from a faulty bearing with an inner race defect and the operating rotational speed is increasing from 13.0 Hz to 25.7 Hz.
• Dataset I-A-3: the vibration data are collected from a faulty bearing with an inner race defect and the operating rotational speed is increasing from 13.5 Hz to 28.5 Hz.
• Dataset I-B-1: the vibration data are collected from a faulty bearing with an inner race defect and the operating rotational speed is decreasing from 24.3 Hz to 9.9 Hz.
• Dataset I-B-2: the vibration data are collected from a faulty bearing with an inner race defect and the operating rotational speed is decreasing from 25.1 Hz to 13.1 Hz.
• Dataset I-B-3: the vibration data are collected from a faulty bearing with an inner race defect and the operating rotational speed is decreasing from 25.8 Hz to 12.0 Hz.
• Dataset I-C-1: the vibration data are collected from a faulty bearing with an inner race defect and the operating rotational speed is increasing from 15.1 Hz to 24.4 Hz, then decreasing to 18.7 Hz.
• Dataset I-C-2: the vibration data are collected from a faulty bearing with an inner race defect and the operating rotational speed is increasing from 14.1 Hz to 23.5 Hz, then decreasing to 18.0 Hz.
• Dataset I-C-3: the vibration data are collected from a faulty bearing with an inner race defect and the operating rotational speed is increasing from 14.8 Hz to 21.7 Hz, then decreasing to 13.6 Hz.
• Dataset I-D-1: the vibration data are collected from a faulty bearing with an inner race defect and the operating rotational speed is decreasing from 25.3 Hz to 14.8 Hz, then increasing to 19.4 Hz.
• Dataset I-D-2: the vibration data are collected from a faulty bearing with an inner race defect and the operating rotational speed is decreasing from 25.3 Hz to 15.1 Hz, then increasing to 19.8 Hz.
• Dataset I-D-3: the vibration data are collected from a faulty bearing with an inner race defect and the operating rotational speed is decreasing from 23.1 Hz to 15.7 Hz, then increasing to 23.6 Hz.
• Dataset O-A-1: the vibration data are collected from a faulty bearing with an outer race defect and the operating rotational speed is increasing from 14.8 Hz to 27.1 Hz.
• Dataset O-A-2: the vibration data are collected from a faulty bearing with an outer race defect and the operating rotational speed is increasing from 12.9 Hz to 23.0 Hz.
• Dataset O-A-3: the vibration data are collected from a faulty bearing with an outer race defect and the operating rotational speed is increasing from 13.3 Hz to 26.3 Hz.
• Dataset O-B-1: the vibration data are collected from a faulty bearing with an outer race defect and the operating rotational speed is decreasing from 24.9 Hz to 9.8 Hz.
• Dataset O-B-2: the vibration data are collected from a faulty bearing with an outer race defect and the operating rotational speed is decreasing from 24.7 Hz to 10.2 Hz.
• Dataset O-B-3: the vibration data are collected from a faulty bearing with an outer race defect and the operating rotational speed is decreasing from 25.4 Hz to 10.3 Hz.
• Dataset O-C-1: the vibration data are collected from a faulty bearing with an outer race defect and the operating rotational speed is increasing from 14.0 Hz to 21.7 Hz, then decreasing to 14.5 Hz.
• Dataset O-C-2: the vibration data are collected from a faulty bearing with an outer race defect and the operating rotational speed is increasing from 14.0 Hz to 24.5 Hz, then decreasing to 19.8 Hz.
• Dataset O-C-1: the vibration data are collected from a faulty bearing with an outer race defect and the operating rotational speed is increasing from 14.2 Hz to 23.4 Hz, then decreasing to 17.6 Hz.
• Dataset O-D-1: the vibration data are collected from a faulty bearing with an outer race defect and the operating rotational speed is decreasing from 26.0 Hz to 18.9 Hz, then increasing to 24.5 Hz.
• Dataset O-D-2: the vibration data are collected from a faulty bearing with an outer race defect and the operating rotational speed is decreasing from 25.2 Hz to 14.9 Hz, then increasing to 19.5 Hz.
• Dataset O-D-3: the vibration data are collected from a faulty bearing with an outer race defect and the operating rotational speed is decreasing from 25.5 Hz to 15.0 Hz, then increasing to 19.6 Hz.
